# Morphofunctional and signaling molecules overlap of the pineal gland and thymus: role and significance in aging

**DOI:** 10.18632/oncotarget.7863

**Published:** 2016-03-02

**Authors:** Michael A. Paltsev, Victoria O. Polyakova, Igor M. Kvetnoy, George Anderson, Tatiana V. Kvetnaia, Natalia S. Linkova, Ekaterina M. Paltseva, Rosa Rubino, Salvatore De Cosmo, Angelo De Cata, Gianluigi Mazzoccoli

**Affiliations:** ^1^ Russian Academy of Science, Moscow, Russian Federation, Russia; ^2^ Department of Pathology, Ott Institute of Obstetrics, Gynecology and Reproductology, St. Petersburg, Russian Federation, Russia; ^3^ Laboratory of Cell Biology and Pathology, Institute of Bioregulation and Gerontology, St. Petersburg, Russian Federation, Russia; ^4^ CRC Scotland and London, United Kingdom; ^5^ Division of Immuhistochemistry, B.V. Petrovsky Russian Surgery Research Center, Moscow, Russian Federation, Russia; ^6^ Department of Medical Sciences, Division of Internal Medicine and Chronobiology Unit, IRCCS Scientific Institute and Regional General Hospital “Casa Sollievo della Sofferenza”, Opera di Padre Pio da Pietrelcina, San Giovanni Rotondo (FG), Italy

**Keywords:** pineal, thymus, melatonin, neuroendocrine-immune, aging, Gerotarget

## Abstract

Deficits in neuroendocrine-immune system functioning, including alterations in pineal and thymic glands, contribute to aging-associated diseases. This study looks at ageing-associated alterations in pineal and thymic gland functioning evaluating common signaling molecules present in both human and animal pinealocytes and thymocytes: endocrine cell markers (melatonin, serotonin, pCREB, AANAT, CGRP, VIP, chromogranin A); cell renovation markers (p53, AIF, Ki67), matrix metalloproteinases (MMP2, MMP9) and lymphocytes markers (CD4, CD5, CD8, CD20). Pineal melatonin is decreased, as is one of the melatonin pathway synthesis enzymes in the thymic gland. A further similarity is the increased MMPs levels evident over age in both glands. Significant differences are evident in cell renovation processes, which deteriorate more quickly in the aged thymus *versus* the pineal gland. Decreases in the number of pineal B-cells and thymic T-cells were also observed over aging. Collected data indicate that cellular involution of the pineal gland and thymus show many commonalities, but also significant changes in aging-associated proteins. It is proposed that such ageing-associated alterations in these two glands provide novel pharmaceutical targets for the wide array of medical conditions that are more likely to emerge over the course of ageing.

## INTRODUCTION

A reduction in the functioning of the nervous, endocrine and immune systems form an appreciable component of aging-associated diseases. Age-associated changes in neuroendocrine and immune networks, particularly age-associated impairments in the functioning of the pineal and thymic glands, are now believed to pay an important role in the mechanisms of aging and the development of age-associated diseases [1-2]. Pinealectomized mice display a decrease in thymic weight, accompanied by cellular depletion and impaired secretory function [3-4]. Pharmacological blockage of the murine pineal gland decreases blood levels of the thymic hormone thymulin. The rhythmic pattern of several immune characteristics associated with glucocorticoid synthesis in the adrenal cortex, a structure tightly associated with pineal gland, is changed in thymectomized mice [5-6]. Such data highlight the reciprocated influences of these glands, with consequences for immune system regulation.

The relationship between the pineal gland and the immune system was established in the second half of the 20th century. After neonatal pinealectomy, the thymus is destroyed and wasting disease develops. The pineal gland hormone, melatonin, stimulates some immune cell processes, increasing T helper (Th)1 lymphocytes and natural killer cells as well as modulating haemopoiesis and immune cell-target cell interactions [7]. Melatonin is also produced by many immune cells, including macrophages, where its autocrine effects can induce an anti-iflammatory M2-phenotype [8] as well as by other systemic and thymic immune cells. Melatonin receptors have also been found in blood and thymic immune cells, indicating the wide immune-regulatory influence of this indole. The first key enzyme in melatonin synthesis, N-acetyltransferase (AANAT), has mature RNA present in the thymus as well as in peripheral blood mononuclear cells [9]. Other work has demonstrated that the human and rat thymus contains melatonin, as well as the mRNA of its two synthesizing enzymes, AANAT and hydroxyindol-O-methyltransferase (HIOMT), the former using serotonin as a precursor to produce N-acetylserotonin, which HIOMT converts to melatonin. When cultured for 24 h rat thymocytes produce high levels of melatonin, leading the authors to propose that not only the rat, but the human, thymus is capable of synthesizing melatonin, which could have intracrine, autocrine and paracrine functions [10]. Indeed, recent work has led to the proposal that all mitochondria-containing cells may produce melatonin [11]. Melatonin also has non-receptor effects and, being amphiphilic, can accumulate around internal organelles, particularly mitochondria, where it can significantly regulate the mitochondrial membrane [12]. Given that mitochondria are crucial to the optimal functioning of thymocytes and pinealocytes, as well as of most other cells, this may be another means by which melatonin may act to co-ordinate functioning in different glands and tissues [13]. It is also of note that about 10% of the pineal gland comprises cytokine-secreting immune cells that are similarly expressed in the thymus, indicating that these two glands may also be subject to similar immune cell regulation, thereby being another means by which their functioning may be co-ordinated. Image analysis of immunocytochemically stained sections of the chicken pineal gland showed that the majority of lymphocytes were CD3+ (80%) with the remaining 20% comprising B-cells and monocytes (Bu-1+). T-cell subsets in the pineal gland included CD4+ (75-80%), CD8+ (20-25%), TCRαβ/Vβ1+ (60%), and TCRγδ+ (15%). All of the T-cell phenotypes were commonly found within the interfollicular septa and follicles of the pineal gland [14]. The plethora of data on the involution of the pineal gland and thymus during aging also suggests a close functional relationship between these organs over the course of aging, with such changes playing a significant role in some of the mechanisms forming the biological underpinnings of aging [15]. Here we studied the morphofunctional and signaling molecules overlap of the pineal gland and thymus during aging.

## RESULTS

### The morphofunctional and signaling molecule similarity of pineal gland and thymus across human age-groups

**Figure 1:**
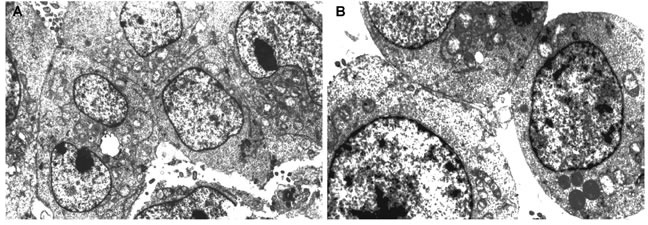
The ultrastructure of pinealocytes and thymic epithelial cells of elderly people evaluated by electron microscopy (1000x) in ultra-thin slices contrasted with uranyl acetate and lead citrate Electron microscopy shows the ultrastructural similarity of pinealocytes **A.** and thymic epithelial cells **B.** in the autopsy samples of elderly people. The cells of both tissues have oval shaped nuclei with heterochromatin and readily perceivable nucleolus. Pinealocytes and thymic epithelial cell contain organelles, including mitochondria and endoplasmic reticulum and a high number of round vesicles, which are likely to consist of biologically active signaling molecules.

Electronic microscopy data show the ultrastructural similarity of pinealocytes and thymic epithelial cells (TEC) in the autopsy samples of elderly people. The cells of both tissues have oval shaped nuclei with heterochromatin and readily perceivable nucleolus. Pinealocytes and TEC have heavy karyolemma. The cytoplasm of both cell types contains a sufficient quantity of organelles, including mitochondria and endoplasmic reticulum (Figure 1). A high number of round vesicles, which can be presumed to consist of biologically active signaling molecules, were evident in both pinealocytes and TEC cytoplasm (Figure 1). Such data may also be taken to indicate that both types of cells can be seen as being within a neuroendocrine-immune system.

### Expression of common signaling molecules in pineal gland and thymus evaluated by immunohistochemistry

**Figure 2:**
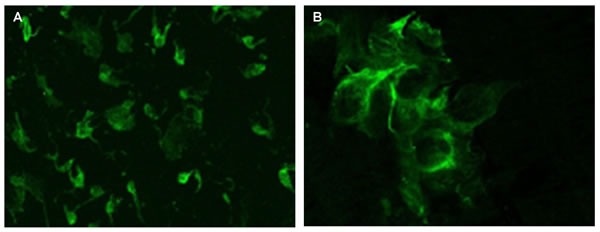
Melatonin expression evaluated by immunofluorescence confocal microscopy (400x) in the pineal gland (A) and thymus (B) of elderly people Samples were fixed in 4% neutral buffered formalin (pH 7.2) for 15 minutes and incubated with primary monoclonal mouse antibodies to melatonin (Mubro Products B.V., 1:50). After washout in phosphate buffer, samples were incubated with a second antibody, rabbit antimouse FITC-conjugated Ig (Dako, 1:100). After washout in phosphate buffer, a medium containing 90% glycerin, 0.02M Tris-HCl (pH 8.0), 0.8% NaN3, and 2% 1.4-di-azabicyclo-(2,2,2)-octan (Sigma-Aldrich) was added to the cells. The preparations were examined under a Leica TCS SP5 confocal microscope using an MRC-1024 system equipped with LaserSharp 5.0 software (Bio-Rad) for confocal image analysis.

**Figure 3:**
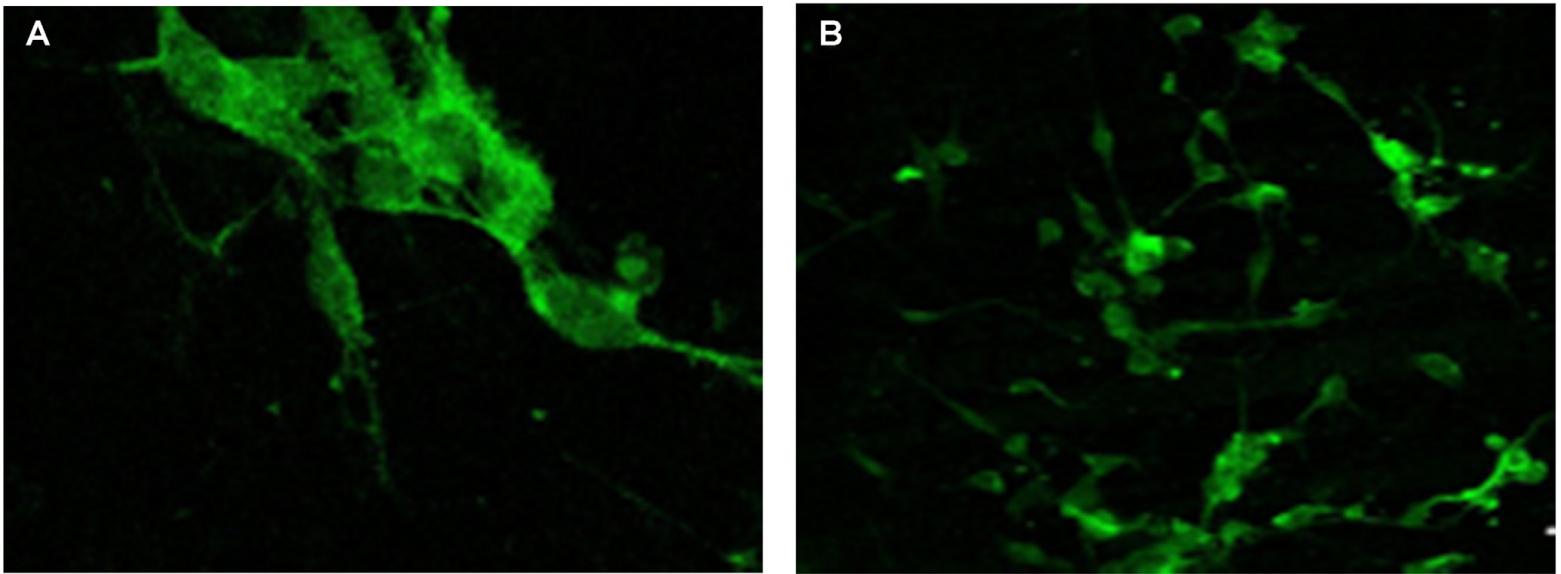
Chromogranin A expression evaluated by immunofluorescence confocal microscopy (400x) in the pineal gland (A) and thymus (B) of elderly people Samples were fixed in 4% neutral buffered formalin (pH 7.2) for 15 minutes and incubated with primary monoclonal mouse antibodies to chromogranin A (Mubro Products B.V., 1:75) for 1 hour. After washout in phosphate buffer, samples were incubated with a second antibody, rabbit antimouse FITC-conjugated Ig (Dako, 1:100). After washout in phosphate buffer, a medium containing 90% glycerin, 0.02M Tris-HCl (pH 8.0), 0.8% NaN3, and 2% 1.4-di-azabicyclo-(2,2,2)-octan (Sigma-Aldrich) was added to the cells. The preparations were examined under a Leica TCS SP5 confocal microscope using an MRC-1024 system equipped with LaserSharp 5.0 software (Bio-Rad) for confocal image analysis.

**Figure 4:**
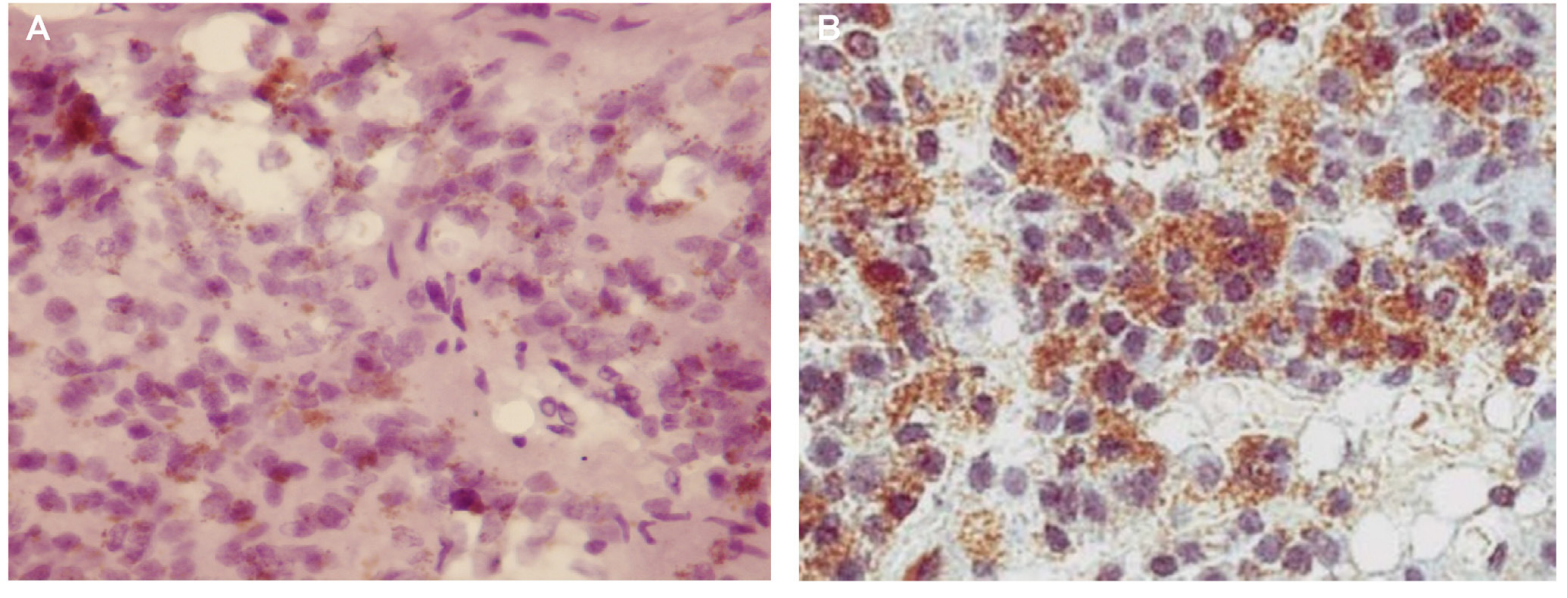
CD5 expression evaluated by immunohistochemistry (200x) in the pineal gland (A) and thymus (B) of elderly people Samples of pineal gland and thymus were fixed in formalin at standard pH, washed with ethanol solutions and fixed using a paraffin-embedding standard technique. Slices with a thickness 3-5 μm were made using a Leica 540M microtome and placed on slides with poly-L-lysine. For histological analysis the samples were stained using the standard hematoxylin-eosin staining technique. The identification of CD5 (1:30, Novocastra) was carried out with an immunohistochemical method using primary mouse monoclonal antibodies, and secondary antibodies, i.e. biotinylated anti-mouse immunoglobulins (Novocastra). Visualization of the reaction was carried out using the avidin-peroxidase complex (ABC-kit) and diaminobenzidine.

By using immunohistochemical methodology, the expression of common signal molecules, including endocrine cells markers (melatonin, serotonin, pCREB, AANAT, CGRP, VIP, chromogranin A), cell renovation markers (p53, AIF, Ki67), MMPs (MMP2, MMP9) and lymphocytes (CD4, CD5, CD8, CD20) can be seen to be evident in the human pineal gland and thymus (Table 1; Figure 2; Figure 3; Figure 4). The expression of melatonin, serotonin, AANAT, pCREB, CGRP, chromogranin A in the pineal gland and thymus decreased over the course of aging, although this was not the case with VIP expression, which showed no aging-associated change. Melatonin and serotonin are hormones that can be synthesized in various tissues of the nervous, immune and endocrine systems. As such, the melatonin and serotonin expression in both pinealocytes and thymic cells indicates the similarity of pineal and thymus glands, suggesting their coordinated regulation and functioning within a neuro-immuno-endocrine system. Moreover, melatonin is a powerful homeostasis regulator in the whole organism, including *via* its synchronization of circadian rhythms, as well as *via* its antioxidant effects and endogenous antioxidant induction that can be coupled to its mitochondria optimizing effects, cytostatic properties and immune modulatory activity. The AANAT enzyme is activated by pCREB, an ATP-dependent transcription factor that is necessary for melatonin synthesis [16, 17]. The aging-associated decrease in pCREB is therefore likely to be intimately linked to a decrease in N-acetylserotonin and therefore melatonin synthesis. It is likely that chromogranin A is stored in the secretory granules and released *via* exocytosis in pinealocytes and thymic cells. At the present time, chromogranin A function still requires clarification. It has been proposed that this water-soluble protein, which consists of 450 amino acids residues, is released into the blood with catecholamines [18]. As such, chromogranin A synthesis and release indicates that the pineal gland and thymus may take part in neuroendocrine regulation at a whole organism level. Other data supports such a neuroendocrine role for these glands, including *via* the secretion of CGRP and VIP [19, 20]. Moreover, CGRP and VIP are also shown to be characterized by decreased expression over the course of aging, which again suggests similar age-related changes arising from the involution of the pineal gland and thymus. Cell renovation processes (indicated by the proliferation/apoptosis ratio) are important indicants of functional activity and organ aging. There are two mechanisms of apoptosis induction: activation of protease-associated intracellular cascade (caspases); and secondly, mitochondrial driven apoptotic effectors. The key proteins in these two overlapping apoptotic pathways are: p53 in caspase-dependent apoptosis; and mitochondrial AIF [21]. Another important signaling molecule is the common cell proliferation marker Ki67 protein, which can be verified in many phases of the cell cycle (G1, G2, S, M) and is absent in quiescent cells in the G0-phase [22, 23]. We show here that AIF-dependent and p53-dependent apoptosis increases in the pineal gland during aging, with both AIF- and p53-dependent apoptosis increasing also in the thymus over aging. However, the expression of the proliferative protein Ki67 decreased over aging only in the thymus of long-lived people. As such, cell renovation processes, as indicated by measures of the proliferation/apoptosis ratio, were maintained at a higher level in the pineal gland *versus* the thymus over the course of aging. The MMPs are the zinc-containing proteins of the extracellular space that participate in cellular activation, differentiation, proliferation, apoptosis and migration, with an important role being played by MMP2 and MMP9 (gelatinases A and B). MMP2 is synthesized by leukocytes and fibroblasts, and breaks down type IV collagen, fibronectin and tenascin-C. MMP9 is produced by macrophages and granulocytes, and, besides breaking down type IV collagen, also hydrolizes elastin [24-26]. MMPs also showed similar changes over aging in the pineal gland and thymus, again indicating a similarity of changes in these two glands. Given that lymphocytes are present in the pineal gland and can produce MMP2, levels of MMP2 may be at least partly determined by the presence of leukocytes. Here it was shown that lymphoid component, which represent 10% of pineal gland tissue, included at least 4 types of cells: CD4+ T-helpers, CD5+ activating pre-T and B-cells, CD8+ cytotoxic T-cells and CD20+ B-lymphocytes. It is likely that the most important of these cells in the pineal gland are B-cells. The quantity of CD4+, CD5+, CD8+ cells in thymus decreased over aging, but the number of thymic B-cells stayed at a constant low level. The pineal gland shows some contrasting results to such leukocyte changes in the thymus, with the levels of pineal CD4+, CD5+, CD8+ cells showing no changes over aging, and the number of pineal B-cells decreasing over aging. As such, B-cells are the most common pineal leukocyte sub-population, with their numbers in this gland decreasing during aging.

**Table 1:** Signaling molecules in pineal gland and thymus of variously aged people

Signaling molecules						
	Pineal gland			Thymus		
	old people	very old people	long-lived people	old people	very old people	long-lived people
Ki67	0.38±0.09	0.54±0.12	0.19±0.03*#	0.58±0.07	0.19±0.03*	0.07±0.02*#
p53	1.15±0.27	1.35±0.08	2.05±0.04*#	4.51±0.11	9.32±0,43*	9.41±0.20*
AIF	4.54±1.15	9.12±1.04*	8.00±1.08*	0.07±0.02	1.35±0.02*	2.61±0.31*#
CGRP	4.49±0.59	5.98±0.74*	3.67±0.25*#	2.38±0.18	0.62±0.17*	0.97±0.15*
MMP2	0.43±0.08	0.22±0.04*	0.11±0.03*#	0.74±0.13	0.21±0.05*	0.16±0.03*
MMP9	0.35±0.09	0.18±0.04*	0.10±0.02*#	0.48±0.11	0.26±0.03*	0.24±0.03*
AANAT	2.21±0.08	1.53±0.06*	0.65±0.04*#	0.53±0.03	0.49±0.04	0.26±0.04*#
pCREB	3.11±0.15	2.16±0.08*	0.97±0.08*#	2.29±0.16	2.04±0.11	1.01±0.04*#
chromogranin A	3.17±0.19	2.07±0.09*	2.09±0.13*	0.67±0.08	0.70±0.06	0.63±0.09
serotonin	3.37±0.21	2.54±0.20*	1.05±0.09*#	0.34±0.03	0.30±0.04	0.09±0.02*#
melatonin	4.56±0.27	2.21±0.11*	0.69±0.08*#	0.29±0.03	0.17±0.02*	0.05±0.01*#
VIP	1.14±0.16	1.11±0.17	1.17±0.12	0.97±0.08	0.86±0.09	0.89±0.08
CD4	0.31±0.07	0.34±0.08	0.71±0.14*#	2.70±0.54	1.58±0.18*	0.32±0.07*#
CD5	2.36±0.44	1.68±0.23	1.11±0.12*#	2.48±0.31	1.66±0.31	1.05±0.12*#
CD8	1.49±0.25	1.70±0.22	2.12±0.32*#	3.88±0.52	3.91±0.49	1.84±0.32*#
CD20	1.52±0.19	0.73±0.21*	1.01±0.19*	0.69±0.12	0.56±0.11	0.65±0.13

* *p*<0.05 as compared to corresponding data of “old people” group,

# *p*<0.05 as compared to corresponding data of “very old people” group; values represent the square ± standard deviation of immunohistochemical expression reported as immunoreaction % area

### Signaling molecules similarity in pinealocyte and thymocyte cultures during cell senescence

**Figure 5:**
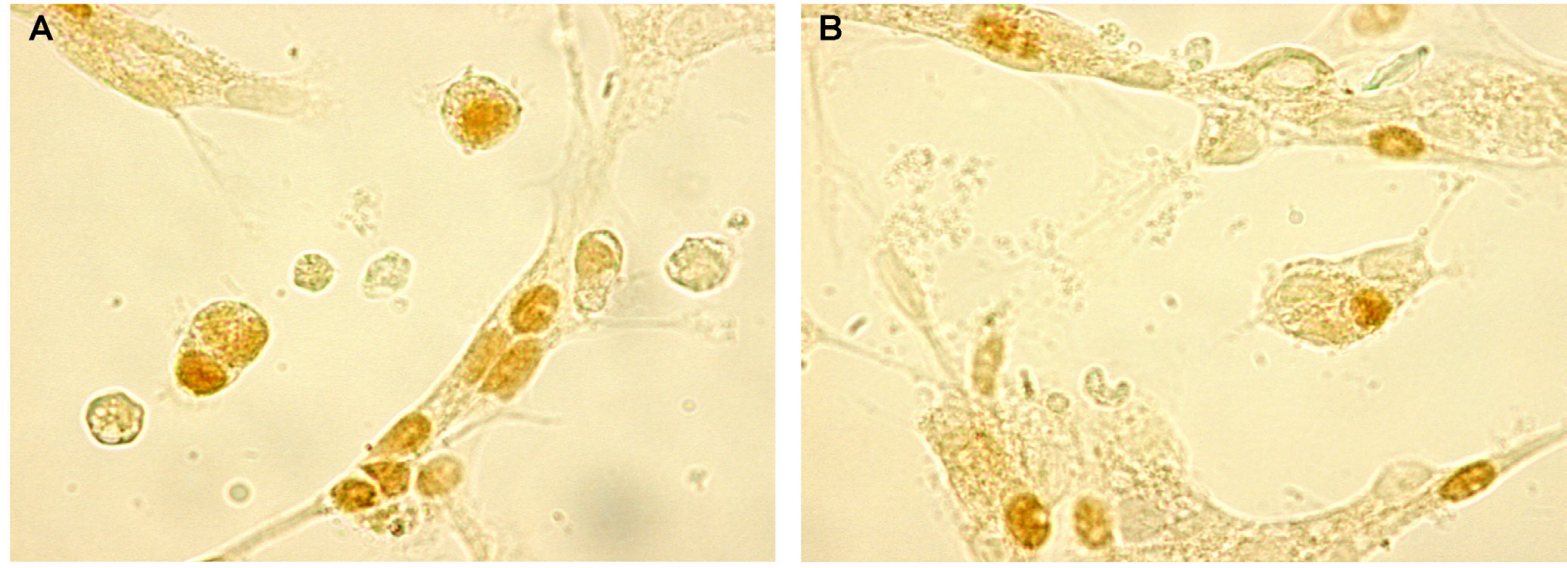
CGRP expression evaluated by immunocytochemistry (200 x) in “young” pinealocytes (A) and thymocytes (B) culture Primary cultures of pinealocytes and thymocytes from 3-month-old Wistar rats were isolated and cultured for the 3^rd^ passage. For immunocytochemistry the primary monoclonal antibodies against CGRP (1:150, Dako) and secondary antibodies biotinylated antimouse immunoglobulins (Novocastra), were used. Permeabilization was carried out using 0.1 % Triton X100. Visualization of the reaction was carried out using the avidin-peroxidase complex (ABC-kit) and diaminobenzidine.

**Figure 6:**
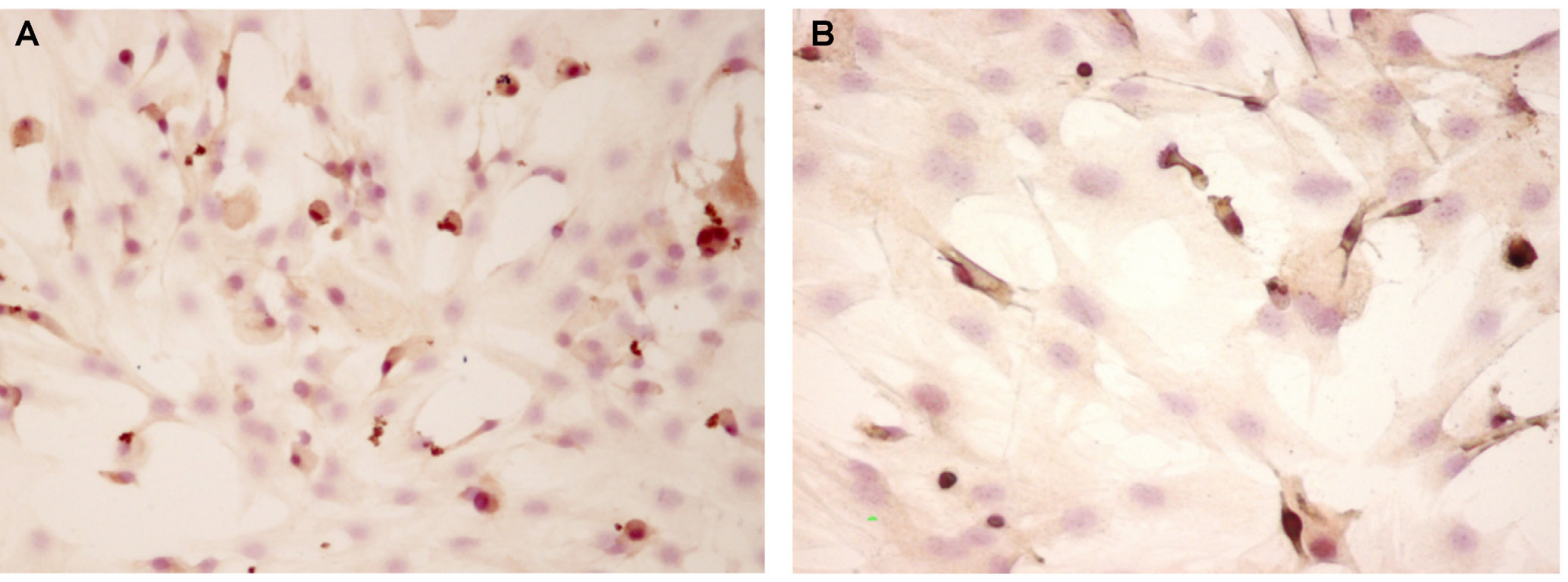
pCREB expression evaluated by immunocytochemistry (200 x) in “young” pinealocytes (A) and thymocytes (B) culture Primary cultures of pinealocytes and thymocytes from 3-month-old Wistar rats were isolated and cultured for the 3^rd^ passage. For immunocytochemistry the primary monoclonal antibodies against pCREB (1:30, Novocastra) and secondary antibodies biotinylated antimouse immunoglobulins (Novocastra), were used. Permeabilization was carried out using 0.1 % Triton X100. Visualization of the reaction was carried out using the avidin-peroxidase complex (ABC-kit) and diaminobenzidine.

**Figure 7:**
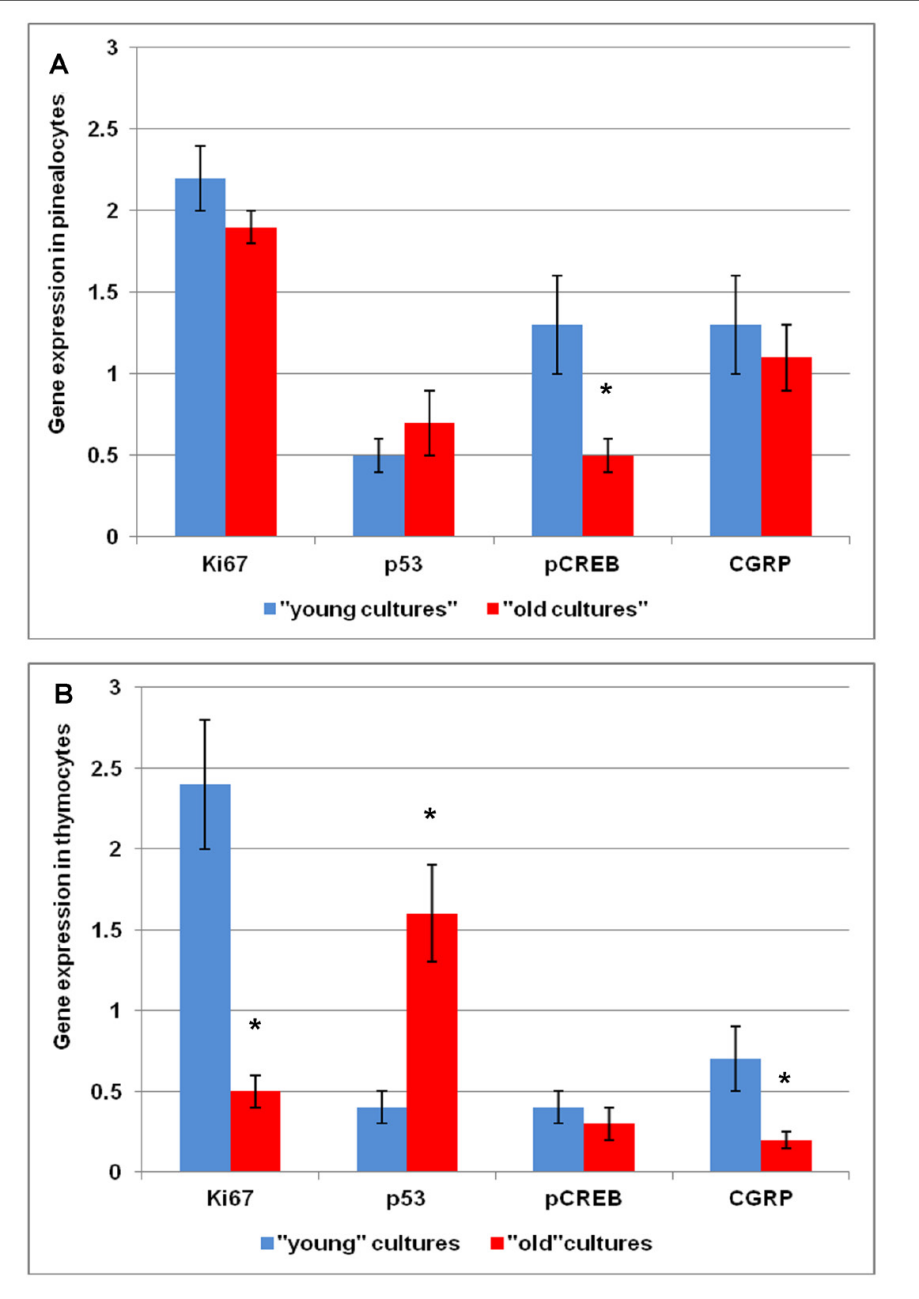
Expression of *MKI67, TP53, CREB, CGRP* genes determined using the Real Time-Polymerase Chain Reaction in pinealocyte cultures (A) and in thymocyte cultures (B) *GAPDH* housekeeping control gene was used to normalize target gene expression levels and the mRNA amount of each target gene relative to *GAPDH* was calculated through the comparative Ct method, also called the 2(−ΔΔCt) method. Three biological replicates were each assayed in triplicate and results were expressed as mean ± standard deviation (SD); * *p* < 0.05 as compared to corresponding data of “young” cell cultures.

This part of the investigation characterized by means of immunocytochemistry and RT-PCR methodology the expression of signaling molecules, including markers of cell renovation (Ki67, p53) and neuroendocrine system function (CGRP, pCREB) (Figure 5 and Figure 6) and their genes (Figure 7), in cell cultures of pinealocytes and thymocytes. It was shown that the Ki67 expression decreased similarly in pinealocyte and thymocyte cell cultures, as in the human autopsy data. At the same time, proapoptotic protein expression increased only in the thymocyte cell cultures (Table 2). The expression of Ki67 and P53 genes did not change in the pinealocyte cell cultures, whereas in the thymocyte cell cultures, simulated aging is characterized by decreased expression of the Ki67 gene (4.8-fold change) and increased expression of the p53 gene (3.6-fold change) (Figure 7). CGRP protein expression in pinealocyte and thymocyte cell cultures decreased very fast over simulated cell aging (Table 2). However, CGRP gene expression is only significantly decreased (6-fold change) in the thymocyte cell cultures (Figure 7). Transcription factor pCREB expression, which plays a key role in melatonin synthesis, was decreased during simulated aging in pinealocyte and thymocyte cell cultures (Table 2). Conversely, pCREB gene expression decreased in pinealocyte cell cultures (2.6-fold change), but its gene expression was not significantly altered in the thymocyte cell cultures (Figure 7).

**Table 2:** Signaling molecules in pineal gland and thymus model of cell cultures senescence

Signaling molecules	Pinealocytes cell cultures		Thymocytes cell cultules	
	“young” cultures	“old” cultures	“young” cultures	“old” cultures
Ki67	0.97±0.06	0.32±0.02*	1.78±0.07	0.53±0.04*
p53	0.45±0.07	0.48±0.05	0.91±0.11	2.12±0.04*
pCREB	1.37±0.06	0.28±0.03*	0.11±0.01	0.04±0.01*
CGRP	3.22±0.07	0.79±0.05*	0.36±0.02	0.12±0.02*

* *p*<0.05 as compared to corresponding data of “young” cell cultures; values represent the square ± standard deviation of immunohistochemical expression reported as immunoreaction % area

## DISCUSSION

Reciprocal influences and bidirectional connections among the nervous, endocrine and immune systems are mediated by shared neuroendocrine hormones, chemo/cytokines and their binding sites, contributing, in turn, to the maintenance of body homeostasis [27]. The neuroendocrine-immune system components interact to modulate immune functioning, in part by influencing cellular immune responses *via* the release of various hormones and neuropeptides into the blood that have direct modulatory action on the immune effectors, or by regulating the hormonal secretion of peripheral endocrine glands [28]. Several studies have reported reciprocal interactions among the pineal gland, thymus and the immune system. Humoral mediators and receptor sites sensitive to signaling molecules mediate the connectivity among these anatomical structures. For instance, melatonin directly stimulates activated T helper (Th)1 cells and natural killer cells, allowing factors influencing circadian and local synthesis of melatonin to impact on the nature of the immune response [13]. As such, the nature of the immune response changes with circadian periodicity, which can be significantly altered over the course of ageing, given that many 24-hour rhythms are changed in old age, including lowering of the night-time melatonin peak [29, 30]. Overall, the circadian system intimately interacts with the neuroendocrine-immune system, potentially at many levels, and aging is associated with changes in neuroendocrine and immune system function [31, 32].

The aim of this study was to evaluate the signaling molecules of the pineal gland and thymus in a group of healthy old, very old and long lived participants. Body homeostasis is maintained by appropriate functioning of the nervous, endocrine and immune system. Such appropriate functioning is determined by the reciprocal interactions among the central neuroendocrine structures (hypothalamus, pituitary, pineal gland), peripheral endocrine glands (adrenal glands, thyroid, gonads), immune tissues (thymus, lymph nodes) and immune competent cells [27]. Many of these interactions are mediated by the release of various hormones and their differential effects at different cells and tissues *via* the activity of hormone binding sites. The pineal gland and thymus may be intimately involved in such neuro-endocrine-immune system interactions. The pineal gland is innervated by post-ganglionic nerve fibers coming from the superior cervical ganglion, which receives fibers from the hypothalamic suprachiasmatic nuclei that are powerfully influenced by light-driven retinal activity *via* the retinohypothalamic tract [27]. As such, alterations in light levels influence activity of the retino-hypothalamic-pineal system, with light dramitically diminishing pineal melatonin production, which may also be regulated by an endogenous free-running pacemaker located in the suprachiasmatic nucleus [27].

Melatonin can modulate immune function by a number of means, including by its powerful antioxidant effects and alpha 7 nicotinic receptor regulation [13, 33], but also *via* its regulation of the opioidergic pathways, leading to the production of opioid agonists and cytokines (IL2 and IL4). Opioid receptors are evident in immunocompetent murine and human cells, with the activation of these cells by antigens or mitogens, leading to the production of opioid peptides, as well as an array of other factors, including ACTH, TSH, GH, IGF-I and VIP. The immunomodulatory role of melatonin may also be mediated by its effects on thymic function, including *via* TRH and TSH, although its ability under experimental conditions to counteract thymic involution, as induced by prednisolone, appears to be thyroid-independent and not correlated to thyroxine levels [34-36].

The data presented here suggest that human pinealocytes and thymic epithelial cells have a similar structure-functional organization and, as the data here indicates, show similar changes over the course of ageing, as evidenced by human autopsy and cell culture data. The data in this study comprised four groups of common signal molecules that were measured in pinealocytes and thymocytes of humans and animals, namely endocrine cell markers (melatonin, serotonin, pCREB, AANAT, CGRP, VIP, chromogranin A), cell renovation markers (p53, AIF, Ki67), MMPs markers (MMP2, MMP9) and lymphocytes markers (CD4, CD5, CD8, CD20). High levels of similarity, and some differences, are evident over age in these two glands. Decreased melatonin and melatonin-related molecule expression were shown to occur in the pineal gland, with decreased AANAT levels also evident in the thymus. The role of local melatonin synthesis over aging in thymic epithelial cells requires investigation, including as to how this could relate to aging-associated immune-senescence. However, cell renovation processes deteriorate more quickly during thymic involution in comparison with aging-associated changes in the pineal gland. A decrease in the number of pineal B-cells and thymic T-cell was observed over aging. A further similarity in age-related changes in these two glands is evident in the increased levels of MMPs over aging.

We have to consider some limitations of the study: *i)* the patients from which the organs were collected succumbed from a wide variety of different conditions that may have impacted the outcome of the study; *ii)* absence of representative samples of the normal aging population, so that the data represent the alterations of pineal gland and thymus in the aging population with some disease; *iii)* lack of reference/control group represented by young subjects, so that it is not possible to state if the pineal gland and thymus of young subjects might show similar patterns.

In conclusion, our study indicates that cellular involution of pineal gland and thymus have different molecular mechanisms, although significant similarities in the aging-associated changes in a number of key proteins and changes. The neuroendocrine system may influence the immune system by releasing various hormones and neuropeptides into the blood with direct immune modulatory actions, or by regulating the hormonal secretion of peripheral endocrine glands, which also exert immune modulating effects. Aging impacts on the functioning of several components of this multi-level structure and their highly complex network of interactions. The data presented here on individual gland changes should provide pharmaceutical targets for the improvement of age-related pathology.

## MATERIALS AND METHODS

The study consisted of two experimental protocols: an autopsy study of pineal and thymic glands; an experimental study of primary cultures of pinealocytes and thymic cells.

### Autopsy pineal and thymic gland samples

Pineal gland and thymus material collected during autopsy from 140 people with ages from 60-100 years was divided into three groups according to the age classification by the World Health Organisation: 1 - old people (60-74 years, *n* = 78, 47 females and 31 males), 2 - very old people (75-89 years, *n* = 42, 33 females and 9 males), and 3 - long-lived people (90 and more years, *n* = 20, 15 females and 5 males). This material was obtained from the Department of Pathology, Ott Institute of Obstetrics, Gynecology and Reproductology, Russian Academy for Medical Sciences, St. Petersburg, Russian Federation. The reason for death of all patients in the 1^st^ and 2^nd^ groups and 17 patients in the 3^rd^ group was related to cardiovascular pathology (myocardial infarction). The cause of death of other 3 patients in the 3^rd^ group was related to sleep apnoea. All patients had arterial hypertension, 4 cases (in the 2^nd^ group) had chronic obstructive lung disease, 2 cases (in the 1^st^ group) and 3 cases (in the 2^nd^ group) had diabetes mellitus. All patients had various ophthalmic pathology - hypermetropia, age-related macular degeneration and glaucoma. No patient had neurodegenerative pathology.

### Electron microscopy

Ultra-thin slices were used for electron microscopy. They were contrasted with uranyl acetate and lead citrate. The study was carried out on the electron microscope JEM-100S (JEOL, Japan). For ultrastructural analysis, whole samples were photographed under a magnification of 14.000. Verification of the secretory granules on the ultrastructural level was made using the uranaffin reaction. For this purpose samples were fixed with 3% glutarate aldehyde on the 0.1M cacodylic buffer (pH = 7.2) for 90 minutes. Subsequently, 1mm cut blocks were rinsed with 0.9% sodium chloride solution 3 times for 15 minutes each. The rinsed blocks were placed into 4% uranyl acetate water solution (pH = 3.9) at 4°C for 18 hours. After rinsing, the blocks were dehydrated in the alcohols and placed in EPON resin.

### Immunohistochemical study

For the immunohistochemical study, samples of pineal gland and thymus were fixed in formalin at standard pH, washed with ethanol solutions and fixed using a paraffin-embedding standard technique. Slices with a thickness 3-5 μm were made using a Leica 540M microtome and placed on slides with poly-L-lysine. For histological analysis the samples were stained using the standard hematoxylin-eosin staining technique. The identification of markers of the signaling molecules serotonin (1:50, Dako), melatonin (1:50, Dako), AANAT (1:150, Dako), phosporylated cyclic AMP-responsive element-binding protein (pCREB) (1:100, Dako), chromogranin A (1:50, Novocastra), vasoactive intestinal peptide (VIP) (1:50, Novocastra), matrix metalloproteinase (MMP)2 (1:60, Novocastra), MMP9 (1:60, Novocastra), p53 (1:30, Novocastra), Ki67 (1:30, Novocastra), apoptosis inducing factor (AIF) (1:100, Dako), calcitonin gene related peptide (CGRP) (1:100, Dako) CD4 (1:30, Novocastra), CD5 (1:30, Novocastra), CD8 (1:30, Novocastra), CD20 (1:30, Novocastra) was carried out with an immunohistochemical method using primary mouse monoclonal antibodies, and secondary antibodies, i.e. biotinylated anti-mouse immunoglobulins (Novocastra). Visualization of the reaction was carried out using the avidin-peroxidase complex (ABC-kit) and diaminobenzidine.

### Immunofluorescence study

For immunofluorescence confocal laser microscopy, samples were placed onto slides. Samples were then fixed in 4% neutral buffered formalin (pH 7.2) for 15 minutes and incubated with primary monoclonal mouse antibodies to melatonin (Mubro Products B.V., 1:50) and chromogranin A (Mubro Products B.V., 1:75) for 1 hour. After washout in phosphate buffer, samples were incubated with a second antibody, rabbit antimouse FITC-conjugated Ig (Dako, 1:100). After washout in phosphate buffer, a medium containing 90% glycerin, 0.02M Tris-HCl (pH 8.0), 0.8% NaN3, and 2% 1.4-di-azabicyclo-(2,2,2)-octan (Sigma-Aldrich) was added to the cells. The preparations were examined under a Leica TCS SP5 confocal microscope using an MRC-1024 system equipped with LaserSharp 5.0 software (Bio-Rad) for confocal image analysis.

### Cell cultures

Primary cultures of pinealocytes and thymic cells were isolated at the Laboratory of Immunology of Aging, St. Petersburg Institute of Bioregulation and Gerontology, Russian Federation. Animals were obtained from the I.P. Pavlov Institute of Physiology, Russian Academy of Sciences, St. Petersburg, Russian Federation. Pineal gland and thymus were dissected from 3-month-old Wistar rats after anesthesia (uretan, 1.1 g/kg of body weight, abdominal injection), cut into small (1 mm) pieces, mashed by scraping with two sterile slide-glasses, and suspended in DMEM containing 10% FBS and penicillin/streptomycin. They were passed through pre-separation filter (Miltenyi Biotech), pelleted by centrifugation at 1500 rpm and re-suspended in DMEM. After that pineal cells were incubated for 45 min at 37°C in DMEM with HEPES (Mediatech, Manassas, VA), supplemented with 6 U/ml papain (Worthington, Freehold, NJ) and 0.2 mg/ml cysteine. Papain was then inactivated by replacing the enzyme solution with complete medium composed of DMEM, 5 mm HEPES, 0.1% Mito^TM^ + serum extender (Collaborative Research, Bedford, MA), 5% heat-inactivated fetal calf serum, 0.75% penicillin-streptomycin-glutamine mix (Invitrogen, Carlsbad, CA). Osmolarity was adjusted to 300 mOsm by addition of distilled water. After centrifugation at 1500 rpm and re-suspension in DMEM thymic cells were cultured in RPMI-1640 growth medium supplemented with 10% FBS, 100U/ml penicillin-streptomycin and 2 mM L-glutamine (Gibco, Carlsbad, CA) and 50 μM 2-mercaptoethanol (Sigma-Aldrich, St. Louis, MO) at 2×10^6^ cells/ml with no treatment or 40 mM Z-VAD(OMe)-FMK (ICN Pharmaceuticals, Aurora, OH) for the indicated amount of time in a humidified chamber with an atmosphere of 5% CO_2_. Thymocyte differentiation in the thymus steps forward through a series of distinct phenotypic stages that correspond to developmental checkpoints. In both human and mouse, thymocytes are in sequence CD4^−^CD8^−^ (double negative, DN), CD4^+^CD3^−^(human immature single positive, ISP) or CD8^+^ CD3^−^(mouse ISP), CD4^+^CD8^+^ (double positive, DP), and finally CD4^+^CD3^+^ or CD8^+^CD3^+^ (single positive, SP). To monitor the synchronized differentiation of large and small pre-selection DP thymocytes *in vitro*, total thymocytes were negatively selected over MACS CD4 MicroBeads (Miltenyi Biotec, Auburn, CA). Flow-through was > 95% DN and ISP thymocytes as determined by flow cytometry, with cells being placed in culture for the indicated amounts of time. Subsequently, every second day, 50% of the culture medium was exchanged for fresh medium. After plating, cells were cultivated for 3^rd^ and 14^th^ passages. The 3rd passage cultures were regarded as “young” cultures, and 14th passage cultures regarded as “old” culture, in compliance with recommendations of the International Association for Cultural Studies (San Francisco, USA). The cells were cultivated in Petri dishes 3.5 cm in diameter, treated with gelatin solution (“Biolot”, Russia). The cells were introduced into the CO2-incubator under standard conditions (5% CO2, 37°C) and into an environment, comprising 15% of Fetal Bovine Serum, 82,5% of iMEM, 1.5% of HEPES-buffer with L-glutamine and 1% penicillin-streptomycin solution added. For cell subcultivation, the Trypsin-Versen solution at the 3:1 ratio was used.

### Immunocytochemistry

For immunocytochemistry the primary monoclonal antibodies against Ki67 (1:30, Novocastra), p53 (1:30, Novocastra), CGRP (1:150, Dako), pCREB (1:30, Novocastra) and secondary antibodies - biotinylated antimouse immunoglobulins (Novocastra) were used. Permeabilization was carried out using 0.1 % Triton X100. Visualization of the reaction was the same as for the autopsied pineal gland and thymus samples.

### Morphometric study

A morphometric study of autopsy material and cell cultures was carried out using the computer analysis system of microscopic images, which consists of the Nikon Eclipse E400 microscope, digital camera Nikon DXM1200, and an Intel Pentium 4 computer with the VideoTest-Morphology 5.2 software (VideoTest, Russia) installed on it. In each case, 10 fields of vision were analyzed at 400x magnification. The surface of the expression was estimated by counting the proportion of immune-positive cells in the total area of the cells in the field of vision. The optical density of the expression was expressed in units. The optical density is a non-dimensional value characterized by the level of light absorption. In our study, the optical density was estimated by VideoTest-Morphology 5.2 to find out the ability of cells to express the investigated markers.

### Real time-polymerase chain reaction

Gene expression studies evaluated those genes known to play an important role in pineal gland and thymus functioning, namely *MKI67*, *TP53*, *CREB*, *CGRP*. Gene expression was determined using the method of Real Time-Polymerase Chain Reaction (RT-PCR). For gene expression studies, the cultures were divided in 2 groups: “young” and “old”, determined as indicated above. RNA stabilization in obtained cell cultures, as well as the following total RNA purification were performed using RNA Protect Cell Reagent and RNeasy Mini Kit (Qiagen, Germany) in accordance with the manufacturer's recommendations. Obtained RNA samples were used to synthesize complementary strands of DNA (first strand synthesis) using oligo (dT)18 (Sintol, Moscow) and reverse transcription kit - Omniscript RT Kit (Qiagen, Germany) in accordance with the manufacturer's recommendations. 1 μg of purified total RNA was used as a matrix in each reverse transcription reaction. Obtained reaction mixture was used as a matrix for PCR in an amount of 1 μl of mixture for 25 μl-volume reaction. Quantitative PCR over intercalate fluorescence dye SYBR Green I was conducted by means of QuantiFast SYBR Green PCR Kit (Qiagen, Germany) and thermocycler CFX96 Real-Time PCR Detection System (BioRad Laboratories, Inc., the USA). Statistical analyses of the results, as well as the diagrams, were executed automatically in the CFX Manager Software. For each cDNA sample at least 3 parallel PCR in nearby cavities (technical parallels) were conducted. Construction of oligonucleotide primers was performed by means of online service NCBI Primer-Blast. For this purpose, pairs of primers were used, one of which complied with regions of two adjoining exons. Corresponding oligonucleotides for the experiment were synthesized (Sintol, Moscow). *GAPDH* housekeeping control gene was used to normalize target gene expression levels and the mRNA amount of each target gene relative to *GAPDH* was calculated through the comparative Ct method, also called the 2(−ΔΔCt) method. Three biological replicates were each assayed in triplicate and results were expressed as mean ± standard deviation (SD).

### Statistical analysis

Hypotheses regarding differences among the values were evaluated by means of the two-sample Wilcoxon rank-sum test and the two-tailed Student's *t*-test, as appropriate. The results are reported as mean ± standard deviation (SD) and values represent the square ± SD of immunohistochemical expression in the old subjects reported as immunoreaction % area. A *p* value < 0.05 was considered statistically significant. All analyses were performed using the STATISTICA 7.0 software (StatSoft, Inc.).
